# Imeglimin-Associated Temporal Changes in γ-Glutamyl Transferase and Total Cholesterol: A Post Hoc Exploratory Analysis of the INFINITY Study

**DOI:** 10.3390/jpm16080433

**Published:** 2026-08-17

**Authors:** Takeshi Osonoi, Shinichiro Shirabe, Miyoko Saito, Mitsuru Hosoya, Satako Douguchi, Kensuke Ofuchi, Makoto Katoh

**Affiliations:** 1 Naka Kinen Clinic, Ibaraki 311-0113, Japan; t-osonoi@kensei-kai.com (T.O.); s-shirabe@kensei-kai.com (S.S.); m-saitou@kensei-kai.com (M.S.); m-hosoya@kensei-kai.com (M.H.); s-douguchi@kensei-kai.com (S.D.); k-ofuchi@kensei-kai.com (K.O.); 2 Division of Research Promotion, Integrated Research Administration Center, Saitama Medical University, Saitama 350-0495, Japan

**Keywords:** imeglimin, γ-glutamyl transpeptidase, total cholesterol, type 2 diabetes mellitus, mitochondrial function, INFINITY Study

## Abstract

**Background:** Imeglimin is a novel oral antidiabetic agent that improves mitochondrial function and glucose metabolism in patients with type 2 diabetes mellitus (T2DM). Its effects on liver enzymes remain unclear. We investigated the effects of imeglimin on hepatic biomarkers, particularly γ-glutamyl transpeptidase (γ-GTP), and the reversibility of these changes after treatment discontinuation. **Methods:** This post hoc analysis of the prospective INFINITY Study included 25 patients with T2DM who completed 6 months of imeglimin treatment followed by a 3-month withdrawal period. Clinical parameters were averaged within predefined 3-month study phases. Changes during treatment and after discontinuation were analyzed. **Results:** Imeglimin significantly improved glycemic control. The geometric mean γ-GTP decreased from 36.2 (27.1–48.4) U/L during the Baseline Phase to 31.1 (23.5–41.1) U/L during the Late Treatment Phase (*p* < 0.05). Aspartate aminotransferase (AST) and alanine aminotransferase (ALT) also showed downward trends, although these changes were not statistically significant. During the Withdrawal Phase, γ-GTP returned to 35.6 (26.4–48.5) U/L (*p* < 0.05 vs. Late Treatment Phase). Patients with baseline γ-GTP >50 U/L showed larger but non-significant changes. Changes during treatment and after discontinuation were inversely correlated (r = −0.480, *p* = 0.015). Only total cholesterol correlated with γ-GTP during both treatment (r = 0.554, *p* = 0.005) and withdrawal (r = 0.450, *p* = 0.024). **Conclusions:** γ-GTP levels showed a significant decrease during imeglimin treatment and increased during the post-treatment observation period in patients with T2DM. Exploratory analyses identified an association between changes in γ-GTP and total cholesterol; however, these findings should be interpreted cautiously and require confirmation in prospective studies with prespecified endpoints.

## 1. Introduction

Type 2 diabetes mellitus (T2DM) is a systemic metabolic disorder associated with a wide range of chronic complications [1]. In recent years, the liver has increasingly been recognized as a primary target organ for diabetes-related metabolic dysfunction, alongside conventional vascular complications [2]. Patients with T2DM frequently exhibit liver enzyme abnormalities accompanied by dyslipidemia, which strongly reflect the underlying insulin resistance and metabolic impairment [3]. Among various hepatic biomarkers, γ-glutamyl transferase (γ-GTP) has traditionally been utilized as an indicator for biliary tract diseases and alcohol-induced liver injury [4]. However, accumulating evidence has demonstrated that elevated γ-GTP levels are closely associated with insulin resistance [5], metabolic syndrome [6], metabolic dysfunction-associated steatotic liver disease (MASLD) [2], cardiovascular disease [7], and all-cause mortality [8]. Therefore, γ-GTP is increasingly recognized as an integrative biomarker reflecting systemic metabolic health rather than merely a marker of hepatic injury.

Given these observations, increasing attention has been directed toward antidiabetic therapies that provide metabolic and hepatoprotective benefits beyond glucose lowering. Several glucose-lowering agents have been reported to improve liver enzyme abnormalities [9]. In particular, glucagon-like peptide-1 receptor agonists (GLP-1RAs) have recently demonstrated efficacy in resolving steatohepatitis and preventing fibrosis progression, leading to regulatory approval for metabolic dysfunction-associated steatohepatitis (MASH) [10]. These advances have heightened interest in the potential hepatic benefits of antidiabetic medications.

Imeglimin is the first-in-class oral antidiabetic agent of the novel glimin class [11]. It improves glycemic control through multiple mechanisms, including optimization of mitochondrial bioenergetics, enhancement of insulin sensitivity, and preservation of pancreatic β-cell function [11,12]. Pivotal clinical trials have demonstrated that imeglimin significantly reduces HbA1c levels and improves glucose metabolism in patients with T2DM [13]. Furthermore, recent studies suggest that imeglimin may exert pleiotropic metabolic benefits mediated by the regulation of mitochondrial function and cellular energy homeostasis, extending beyond its primary glucose-lowering efficacy [14].

As a prominent clinical evaluation of these pleiotropic effects, the INFINITY Study—an exploratory, prospective interventional trial—demonstrated that imeglimin extends erythrocyte lifespan, which potentially leads to disproportionately higher HbA1c levels relative to the actual glycemic status [15,16]. Furthermore, a post hoc analysis of the same study suggested that imeglimin improves erythrocyte deformability [17]. These findings indicate that imeglimin holds therapeutic promise for mitigating chronic diabetic complications, thereby expanding its clinical utility beyond conventional glycemic control [17]. Although several glucose-lowering agents have been reported to improve liver enzymes [9], the effects of imeglimin on γ-GTP and how these changes relate to metabolic indicators remain to be fully elucidated. In particular, whether the changes in γ-GTP observed during imeglimin therapy represent a reversible phenomenon, and whether these changes are associated with fluctuations in lipid metabolism, have not yet been investigated.

To address these gaps, the present study conducted a post hoc analysis using data from the exploratory, prospective interventional “INFINITY Study” to evaluate the detailed dynamics of γ-GTP during imeglimin therapy and after its discontinuation in patients with type 2 diabetes mellitus. The primary objective of this study was to verify the reversibility of imeglimin-induced γ-GTP changes and to comprehensively investigate their associations with various metabolic parameters—including glycemic status, lipid profiles, body mass index, and other liver enzymes—thereby identifying specific metabolic markers that are intrinsically linked to γ-GTP fluctuations.

## 2. Materials and Methods

### 2.1. Study Design

The present study was a post hoc analysis of the INFINITY Study, a prospective, single-arm, open-label, exploratory clinical trial conducted at Naka Kinen Clinic, Japan. Participants received imeglimin (TWYMEEG^®^) at a dose of 1000 mg twice daily for 6 months. A 2-month pre-observation period was incorporated to establish baseline measurements, followed by a 3-month post-treatment follow-up period to evaluate the reversibility of treatment-related changes. Throughout the pre-observation, treatment, and follow-up periods, initiation of new antidiabetic medications or dose escalation of existing therapies was not permitted. However, participants receiving stable doses of metformin and/or α-glucosidase inhibitors were allowed to continue these medications without modification [15].

### 2.2. Participants

**Figure 1:**
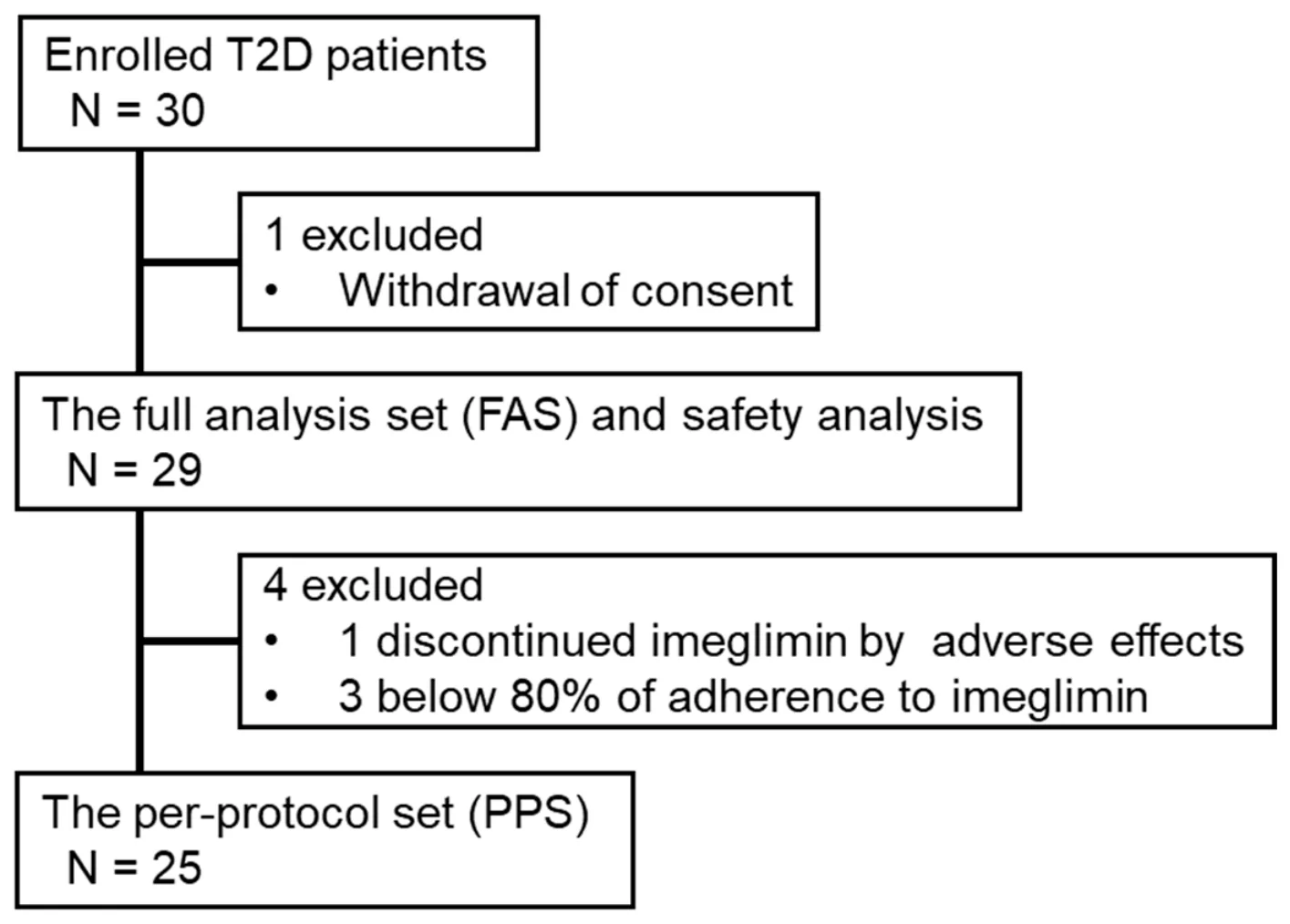
CONSORT-style flow diagram in the INFINITY study. Adapted from [16].

Eligible participants were adults with type 2 diabetes mellitus (T2DM) who were either treatment-naïve or receiving stable treatment with metformin and/or α-glucosidase inhibitors before initiation of imeglimin. At baseline, glucose-lowering medications included α-glucosidase inhibitors alone (31.0%), metformin alone (24.1%), and their combination (3.4%), while 41.4% of participants were treatment-naïve, as previously reported. A total of 30 patients were initially enrolled in the INFINITY Study. One participant withdrew consent, resulting in a full analysis set (FAS) of 29 patients. Subsequently, four patients were excluded from the per-protocol set (PPS) of 25 patients because of treatment discontinuation or poor treatment adherence. One of these participants discontinued imeglimin at Month 1 because of gastrointestinal adverse effects. For the present post hoc analysis, data from the PPS population (*n* = 25) were used to evaluate longitudinal changes in liver enzymes, lipid parameters, and metabolic indices during imeglimin treatment and after drug withdrawal. The participant flow is shown in Figure 1 (CONSORT-style flow diagram adapted for this single-arm trial) [16].

### 2.3. Clinical and Laboratory Assessments

Venous blood samples were collected during four predefined study phases: the Baseline Phase (months −2 to 0), Early Treatment Phase (months 1 to 3), Late Treatment Phase (months 4 to 6), and Withdrawal Phase (months 7 to 9). At the screening visit (month −2), blood sampling was performed under non-fasting conditions to assess study eligibility. Therefore, fasting-dependent parameters, including fasting blood glucose (FBG) and fasting triglycerides (FTGs), were not obtained at this time point. Baseline values for these parameters were established using measurements obtained after an overnight fast of at least 10 h from month −1 onward. All blood samples collected during the treatment and withdrawal phases were obtained after an overnight fast of at least 10 h.

Routine laboratory analyses were performed using standard automated analyzers at Naka Kinen Clinic. Glycemic parameters included FBG, glycated hemoglobin (HbA1c), and glycoalbumin (GA). Liver-related parameters included γ-glutamyl transpeptidase (γ-GTP), aspartate aminotransferase (AST), and alanine aminotransferase (ALT). Lipid parameters included total cholesterol (T-CHO), high-density lipoprotein cholesterol (HDL-C), and FTGs. Anthropometric measurements were obtained at each study visit. Height measured at month −2 was used throughout the study, whereas body weight was assessed at each visit. Body mass index (BMI) was calculated as weight divided by the square of height (kg/m^2^).

To assess insulin resistance and hepatic fibrosis, the following metabolic indices were calculated:

Triglyceride–Glucose (TyG) Index:

The TyG index was calculated using the following formula:

TyG index = ln [FTGs (mg/dL) × FBG (mg/dL)/2]

Fibrosis-4 (FIB-4) Index:

The FIB-4 index was calculated using the following formula:

FIB-4 index = [Age (years) × AST (U/L)]/[Platelet count (10^9^/L) × √ALT (U/L)].

### 2.4. Statistical Analysis

All analyses were performed using the PPS population (n = 25). Laboratory and clinical parameters obtained at monthly visits were averaged within four predefined study phases: Baseline Phase (months −2 to 0), Early Treatment Phase (months 1 to 3), Late Treatment Phase (months 4 to 6), and Withdrawal Phase (months 7 to 9). Data are presented as mean ± standard deviation (SD) unless otherwise specified. Because hepatic enzymes (γ-GTP, AST, and ALT), FTGs, and FIB-4 index exhibited skewed distributions, these variables were logarithmically transformed using the base-10 logarithm (log10) before statistical analyses. For presentation in tables and figures, log-transformed variables were back-transformed and expressed as geometric means with corresponding 95% confidence intervals (CIs) [lower and upper limits]. Longitudinal changes in continuous variables were analyzed using mixed-effects models for repeated measures, with study phase as a fixed effect and participant as a random effect. An unstructured covariance matrix was used to account for within-subject correlations arising from repeated measurements. Prespecified pairwise comparisons were performed between Baseline and Early Treatment, Baseline and Late Treatment, and Late Treatment and Withdrawal using Tukey-adjusted contrasts. These longitudinal mixed-effects analyses constituted the primary statistical analyses of the present study.

To explore interindividual temporal patterns of change during treatment and after drug withdrawal, two exploratory indices were predefined:

- Change during treatment (ΔTreatment): Late Treatment Phase − Baseline Phase
- Change after discontinuation (ΔWithdrawal): Withdrawal Phase − Late Treatment Phase

Associations between ΔTreatment and ΔWithdrawal were analyzed as exploratory analyses to describe interindividual patterns of change over time. Because these two change scores share the Late Treatment measurement, the analyses may be affected by mathematical coupling and were therefore considered hypothesis-generating. The findings were interpreted in conjunction with the primary longitudinal mixed-effects analyses and were not used to establish treatment reversibility or causal mechanisms.

Pearson’s correlation coefficients (r) and simple linear regression analyses were used to evaluate the following:

- The relationships between ΔTreatment and ΔWithdrawal for each clinical parameter.
- Associations between changes in γ-GTP and concurrent changes in metabolic and clinical parameters. Specifically, correlations were analyzed separately for treatment-phase changes (ΔTreatment) and withdrawal-phase changes (ΔWithdrawal).

To investigate the influence of baseline liver enzyme status, participants were stratified according to baseline γ-GTP levels into a high γ-GTP group (>50 U/L, n = 8) and a low γ-GTP group (≤50 U/L, n = 17).

All statistical analyses were performed using GraphPad Prism version 8.4.3 (GraphPad Software LLC, San Diego, CA, USA). A two-sided *p* value < 0.05 was considered statistically significant. Because these analyses were exploratory and hypothesis-generating, nominal *p* values were interpreted cautiously and were not considered confirmatory evidence of specific biological associations.

## 3. Results

### 3.1. Baseline Characteristics

A total of 25 patients (20 males) with T2DM were included in the per-protocol analysis. The mean age was 63.5 ± 11.4 years, and the mean duration of diabetes was 5.3 ± 5.0 years. At baseline (0 months), the clinical characteristics were as follows: BMI, 25.4 ± 3.1 kg/m^2^; FBG, 146.0 ± 23.6 mg/dL; HbA1c, 7.5 ± 0.5%; GA, 18.7 ± 2.3%; γ-GTP, 36.1 (26.9–48.4) U/L; AST, 22.6 (19.5–26.1) U/L; ALT, 23.9 (18.8–30.5) U/L; T-CHO, 191.4 ± 30.8 mg/dL; HDL-C, 57.2 ± 11.9 mg/dL; FTGs, 120.4 (102.1–141.9) mg/dL; TyG Index, 8.98 ± 0.44; and FIB-4 Index, 1.48 (1.18– 1.86). The T2DM patients in the study had hypertension (68.0%), dyslipidemia (88.0%), and cerebrovascular or cardiovascular disease (4.0%).

### 3.2. Longitudinal Changes in Clinical, Metabolic, and Liver-Related Parameters Across the Study Phases

Table 1 summarizes the longitudinal changes in clinical, metabolic, and liver-related parameters across the four predefined study phases in the PPS (n = 25). Among the glycemic parameters, FBG, HbA1c, and GA were significantly reduced during both the Early Treatment Phase (all *p* < 0.01) and the Late Treatment Phase (*p* < 0.05 or *p* < 0.01) compared with the Baseline Phase. These improvements were attenuated after imeglimin withdrawal, and values returned to levels comparable to those observed during the Baseline Phase in the Withdrawal Phase (all *p* < 0.01). No significant changes in BMI were observed throughout the study period (*p* = 0.450).

**Figure 2:**
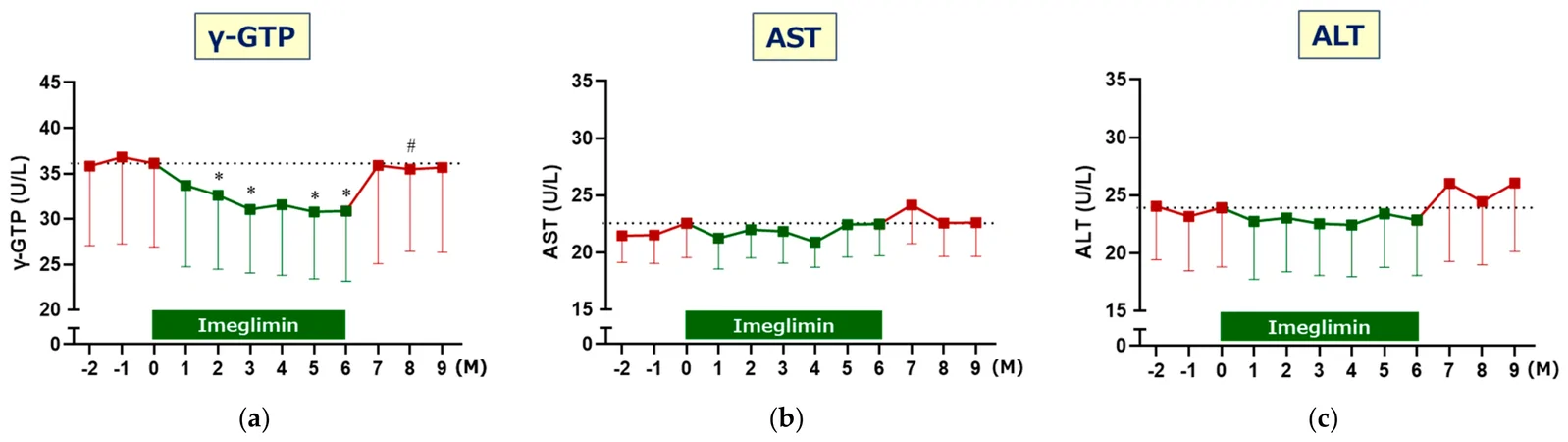
Longitudinal changes in serum liver enzymes, (**a**) γ-GTP, (**b**) AST, and (**c**) ALT, during and after imeglimin treatment. The green bar represents the 6-month imeglimin treatment period. The dashed horizontal line represents the baseline reference (0% change relative to Month 0). Values are presented as geometric means. Only the lower 95% CIs are shown for graphical clarity. * *p* < 0.05 vs. Month 0; ^#^ *p* < 0.05 vs. Month 6. Statistical comparisons were performed using mixed-effects analysis followed by Tukey’s multiple comparisons test.

For the liver-related parameters, geometric means calculated from log-transformed values demonstrated significant reductions in γ-GTP during both the Early Treatment Phase [32.4 (24.5–42.9) U/L] and the Late Treatment Phase [31.1 (23.5–41.1) U/L] compared with the Baseline Phase [36.2 (27.1–48.4) U/L] (both *p* < 0.05). Following imeglimin withdrawal, γ-GTP increased to 35.6 (26.4–48.5) U/L during the Withdrawal Phase, returning to levels comparable to those observed during the Baseline Phase (*p* < 0.05 versus the Late Treatment Phase) (Figure 2). In contrast, AST and ALT showed similar trends but did not change significantly throughout the study period (*p* = 0.249 and *p* = 0.101, respectively) (Figure 2). To facilitate interpretation of the magnitude of these changes, treatment effects were expressed as geometric mean ratios (GMRs) obtained by back-transforming the estimated differences from the mixed-effects model fitted to log10-transformed γ-GTP values. Compared with the Baseline Phase, γ-GTP during the Late Treatment Phase was 14.2% lower (GMR 0.858, 95% CI 0.753–0.976; nominal *p* = 0.0162). Following imeglimin discontinuation, γ-GTP increased by 14.6% relative to the Late Treatment Phase (GMR 1.146, 95% CI 1.004–1.309; nominal *p* = 0.0412). No significant difference was observed between the Withdrawal and Baseline phases (GMR 0.983, 95% CI 0.861–1.124; nominal *p* = 0.9849), indicating that γ-GTP returned to levels comparable to those observed at baseline. A sensitivity analysis using the Full Analysis Set (FAS, N = 29) showed consistent results for γ-GTP, AST, and ALT (Supplemental Figure S1). The direction and statistical significance of γ-GTP changes were consistent between the PPS and FAS analyses. No significant changes were observed in lipid parameters (T-CHO, HDL-C, and FTGs) or the FIB-4 index across the study phases. However, the TyG index tended to decrease during the treatment period and increased significantly during the Withdrawal Phase compared with the Late Treatment Phase (*p* < 0.01).

### 3.3. Influence of Baseline γ-GTP Levels on Reversible γ-GTP Responses

**Figure 3:**
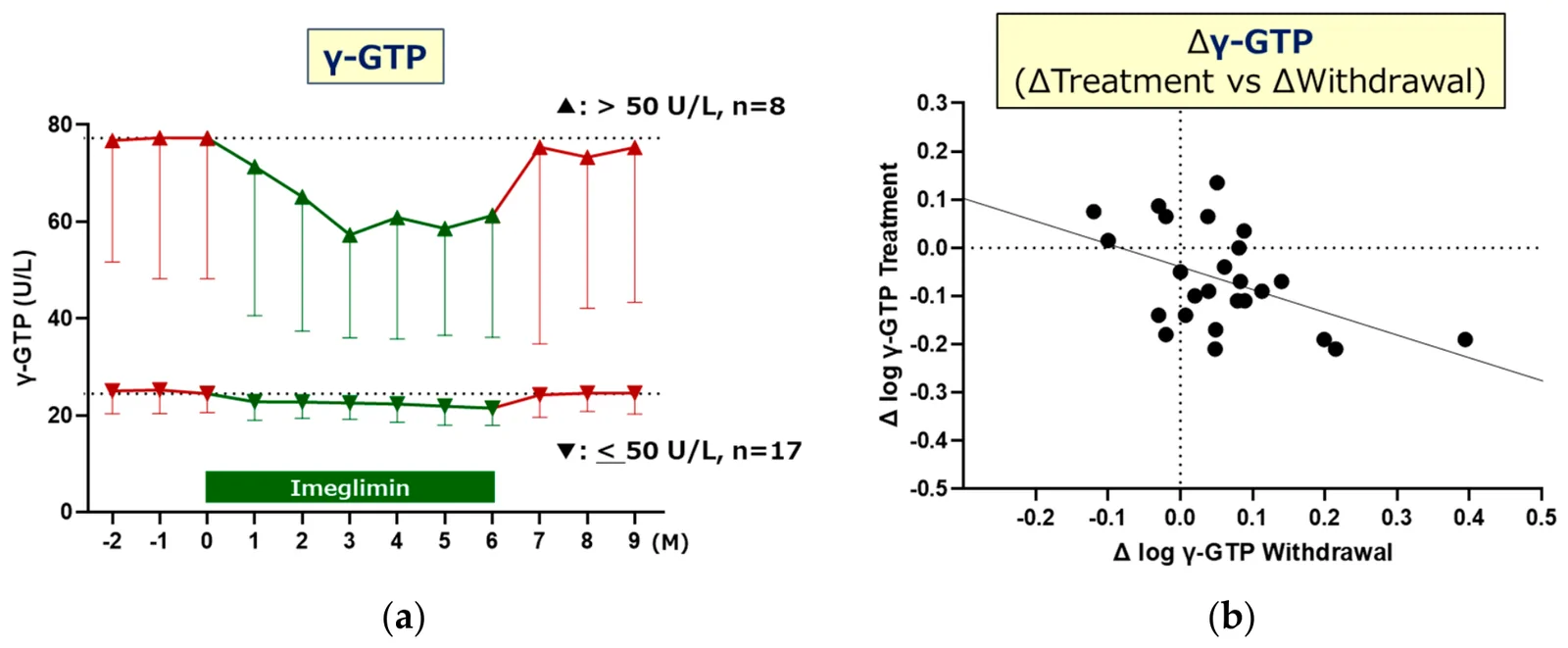
Reversible changes in γ-GTP according to baseline γ-GTP levels and the association between change during treatment and change after discontinuation. (**a**) Longitudinal changes in γ-GTP stratified by baseline γ-GTP levels (>50 U/L and ≤50 U/L). The green bar represents the 6-month imeglimin treatment period. The dashed horizontal line represents the baseline reference (0% change relative to Month 0). Values are presented as geometric means. Only the lower 95% CIs are shown for graphical clarity. (**b**) Correlation between γ-GTP change during treatment (ΔTreatment) and change after discontinuation (ΔWithdrawal). Change during treatment was defined as the change from the Baseline Phase to the Late Treatment Phase, whereas change after discontinuation was defined as the change from the Late Treatment Phase to the Withdrawal Phase. γ-GTP values were log10-transformed before analysis. Pearson’s correlation coefficient (r) and *p* values are shown.

To further characterize the reversible changes in γ-GTP, participants were stratified according to baseline γ-GTP levels into a high γ-GTP group (>50 U/L, n = 8) and a low γ-GTP group (≤50 U/L, n = 17). Participants with baseline γ-GTP levels >50 U/L tended to exhibit greater reductions in γ-GTP during imeglimin treatment and greater increases following drug withdrawal than those with baseline γ-GTP levels ≤50 U/L (Figure 3a).

Among the 25 participants, 16 reported habitual alcohol consumption and 9 did not. Comparable reversible γ-GTP responses were observed in participants with and without habitual alcohol consumption (Supplemental Figure S2).

Consistent with these findings, a significant inverse correlation was observed between the changes during treatment and after discontinuation of log-transformed γ-GTP (*r* = −0.480, *p* = 0.015; Figure 3b), indicating that participants with greater reductions in γ-GTP during imeglimin treatment tended to exhibit greater increases following drug withdrawal.

### 3.4. Exploratory Analyses of Interindividual Response Patterns

To explore interindividual patterns of change beyond the average longitudinal effects estimated by the mixed-effects models, associations between individual changes during treatment and after discontinuation were examined (Table 2). For γ-GTP, an inverse association between ΔTreatment and ΔWithdrawal was observed (Section 3.2). Similar inverse associations were observed for total cholesterol (T-CHO; r = −0.631, *p* < 0.001), log-transformed fasting triglycerides (FTGs; r = −0.491, *p* = 0.013), the TyG index (r = −0.448, *p* = 0.025), and BMI (r = −0.458, *p* = 0.021). No significant associations were observed for FBG, HbA1c, GA, AST, ALT, or the FIB-4 index. These analyses were exploratory and were intended only to describe individual temporal response patterns.

### 3.5. Associations Between γ-GTP Responses and Corresponding Changes in Metabolic Parameters

**Figure 4:**
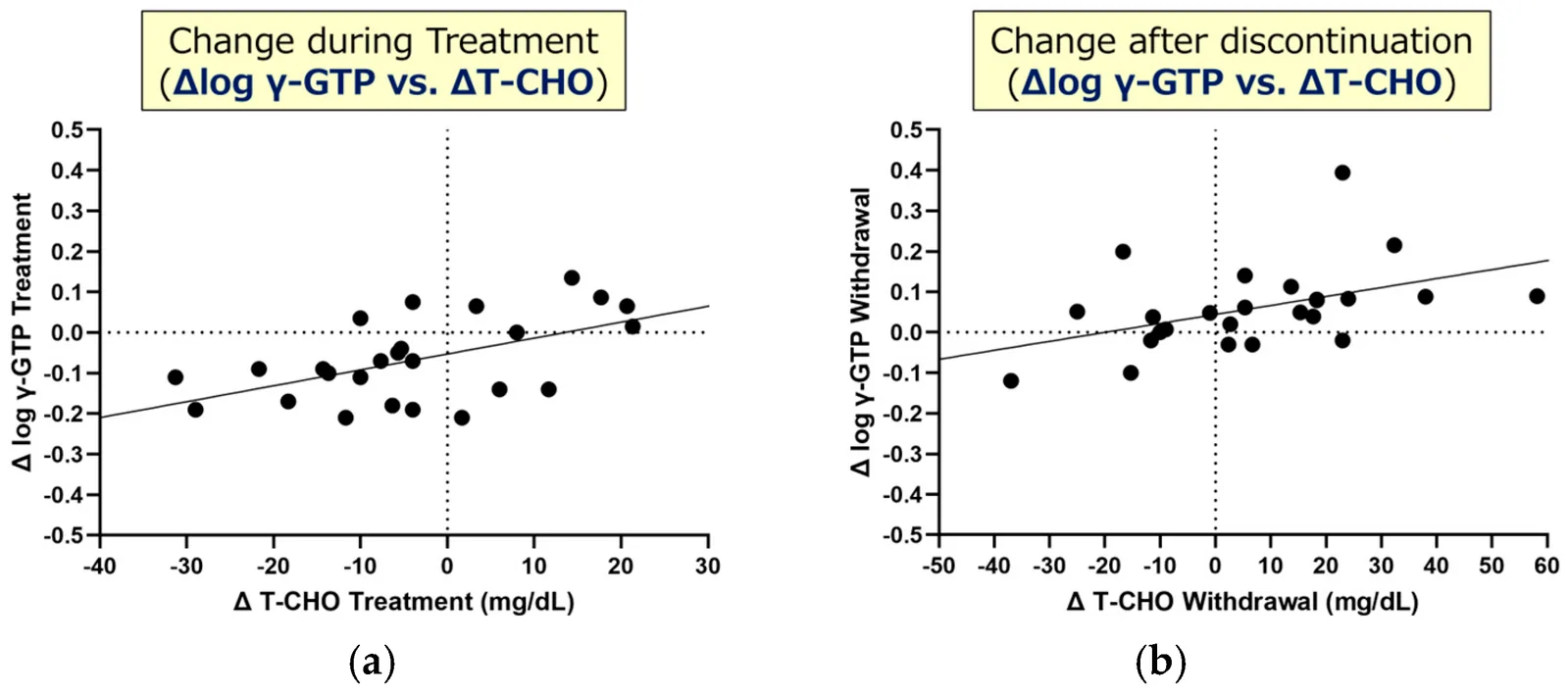
Associations between changes in γ-GTP and total cholesterol during the treatment and withdrawal phases. (**a**) Correlation between the change during treatment of log10-transformed γ-GTP (ΔTreatment) and the corresponding change in total cholesterol (ΔT-CHO). (**b**) Correlation between the change after discontinuation of log10-transformed γ-GTP (ΔWithdrawal) and the corresponding change in total cholesterol (ΔT-CHO). Change during treatment was defined as the change from the Baseline Phase to the Late Treatment Phase, and change after discontinuation as the change from the Late Treatment Phase to the Withdrawal Phase. Pearson’s correlation coefficients (r) and corresponding *p* values are shown. γ-GTP was analyzed after log10 transformation.

To identify metabolic factors associated with reversible γ-GTP changes, correlations between γ-GTP responses and the corresponding changes in clinical and metabolic parameters were examined (Table 3). For the change during treatment, log-transformed γ-GTP was significantly correlated with changes in total cholesterol (ΔT-CHO; r = 0.554, *p* = 0.005) (Figure 4a) and BMI (ΔBMI; r = 0.449, *p* = 0.024). For the change after discontinuation, log-transformed γ-GTP was significantly correlated with the corresponding change in total cholesterol (ΔT-CHO; r = 0.450, *p* = 0.024) (Figure 4b). No significant associations were observed with BMI (ΔBMI; r = 0.355, *p* = 0.081), log-transformed fasting triglycerides (r = 0.109, *p* = 0.605), the TyG index, or the other clinical parameters during the withdrawal phase. Among all parameters examined, total cholesterol was the only variable that showed significant associations with γ-GTP during both the treatment and withdrawal phases.

## 4. Discussion

### 4.1. Principal Findings

This post hoc analysis of the prospective INFINITY Study evaluated longitudinal changes in liver-related biochemical parameters following imeglimin treatment in patients with T2DM. First, γ-GTP levels were significantly lower during the treatment period, whereas AST and ALT showed only non-significant downward trends. Second, γ-GTP levels increased after treatment discontinuation and approached baseline levels during the withdrawal phase. Third, among the exploratory correlations examined, total cholesterol showed an association with γ-GTP changes during both treatment and withdrawal; however, this finding should be interpreted cautiously. Patients with higher baseline γ-GTP levels also tended to show greater reductions during treatment, although this trend did not reach statistical significance. Collectively, these findings suggest that γ-GTP changes may not be fully explained by changes in conventional glycemic parameters alone. The observed changes in γ-GTP and total cholesterol raise the possibility that imeglimin may influence hepatic metabolic pathways; however, whether these biochemical changes have clinical relevance requires further investigation. These analyses were performed as post hoc exploratory analyses because the favorable change in γ-GTP was unexpectedly observed during safety evaluation. Therefore, the findings should be interpreted as hypothesis-generating observations rather than confirmatory evidence.

### 4.2. Potential Clinical Significance of the γ-GTP-Lowering Effect of Imeglimin

γ-GTP has traditionally been regarded as a biomarker of cholestatic liver disease and alcohol-related liver injury [4]. However, accumulating evidence indicates that γ-GTP also reflects systemic metabolic dysfunction rather than merely hepatocellular injury [2,5,6,7,8] and is increasingly recognized as a marker of disturbances in hepatic redox homeostasis, oxidative stress, and metabolic regulation [18,19]. Against this background, the reversible reduction in γ-GTP observed during imeglimin therapy suggests that the drug may exert favorable effects on hepatometabolic pathways beyond its glucose-lowering action, possibly through improvements in mitochondrial function and redox homeostasis.

Although the present study was not designed to evaluate clinical outcomes, the observed reduction in γ-GTP may have implications extending beyond liver biochemistry. Collectively, these findings support the concept that the pleiotropic actions of imeglimin may extend beyond glucose lowering and warrant further investigation into its potential role in preventing or ameliorating metabolic liver disease and other diabetes-related organ complications.

### 4.3. Reversible γ-GTP Changes and Their Association with Total Cholesterol

Although several clinical parameters exhibited a similar temporal pattern, with modest decreases during the treatment phase followed by increases after imeglimin withdrawal at the group level, this alone does not necessarily indicate a coordinated pharmacological response. To further characterize individual temporal patterns of change, we evaluated the relationship between change during treatment and changes after withdrawal for each parameter. These correlation analyses were performed as exploratory analyses to characterize interindividual temporal response patterns in conjunction with the primary longitudinal mixed-effects analyses. Because these change indices share the Late Treatment measurement, the results should be interpreted as hypothesis-generating rather than confirmatory evidence of treatment reversibility. Among the parameters examined, significant inverse correlations were observed only for BMI, total cholesterol, fasting triglycerides, and the TyG index in addition to γ-GTP. In contrast, glycemic parameters, including FBG, HbA1c, and glycoalbumin, did not demonstrate such individual temporal associations despite showing significant improvements during treatment. This finding suggests that the magnitude of changes after imeglimin withdrawal varied considerably among individuals, potentially reflecting interindividual differences in residual β-cell function, insulin sensitivity, or lifestyle factors [11,20]. By contrast, γ-GTP showed a relatively consistent pattern of change during treatment and after withdrawal in this exploratory analysis. Although this observation should be interpreted cautiously, it suggests that γ-GTP may warrant further investigation as a marker of individual metabolic responses during imeglimin treatment and withdrawal. Further studies are required to determine whether γ-GTP represents a specific hepatometabolic response to imeglimin or reflects other metabolic adaptations associated with treatment.

Another important finding was the consistent association between γ-GTP and total cholesterol in the exploratory analyses. Among the parameters that demonstrated coordinated temporal response patterns, as evidenced by significant inverse correlations between individual changes during treatments and after discontinuation, only total cholesterol was significantly associated with γ-GTP during both the treatment and withdrawal phases. BMI showed a modest trend toward association during the withdrawal phase, whereas fasting triglycerides and the TyG index, despite exhibiting similar temporal response patterns, were not significantly associated with γ-GTP. These observations raise the possibility that changes in γ-GTP may be more closely related to cholesterol metabolism than to the other metabolic parameters examined in the present study [21]. However, because these findings were derived from exploratory analyses, they should be interpreted cautiously. Although the consistency of the association across both treatment and withdrawal phases is noteworthy, further prospective studies with prespecified analyses and mechanistic assessments are required to determine whether this relationship reflects a biologically meaningful link between γ-GTP, cholesterol homeostasis, and the metabolic effects of imeglimin [21,22].

### 4.4. Potential Mechanisms and Future Perspectives

Imeglimin is distinguished from conventional glucose-lowering agents by its direct effects on mitochondrial bioenergetics [11,12]. Experimental studies have demonstrated that imeglimin improves mitochondrial function, reduces excessive reactive oxygen species production, and restores cellular energy homeostasis [14,23]. Consistent with the present findings, Tajima et al. recently reported significant reductions in serum γ-GTP during imeglimin treatment together with improvements in insulin sensitivity and oxidative stress markers, suggesting that the metabolic effects of imeglimin may be mediated, at least in part, through improvements in mitochondrial function and redox homeostasis [24]. Because γ-GTP plays a key role in glutathione metabolism and has been associated with oxidative stress [4,18,19], the reversible reduction in γ-GTP observed in the present study may reflect changes in serum biochemical parameters associated with metabolic status. Unlike the study by Tajima et al. [24], which evaluated only the treatment period, the planned withdrawal phase incorporated into the present study enabled assessment of the temporal reversibility of γ-GTP changes. The observed association between γ-GTP and total cholesterol raises the possibility [21,22] that mitochondrial regulation by imeglimin may be linked to hepatic cholesterol metabolism, although this interpretation remains speculative and requires mechanistic confirmation.

These findings may also have potential clinical implications. Given the growing recognition of γ-GTP as a marker of systemic metabolic dysfunction, longitudinal changes in γ-GTP may provide complementary information regarding metabolic responses during imeglimin therapy, particularly in patients with T2DM accompanied by metabolic liver dysfunction [2,5,6]. In addition, the exploratory observation that participants with higher baseline γ-GTP levels tended to exhibit greater reductions during treatment warrants further investigation but should not be interpreted as evidence of differential treatment benefit. Future prospective studies incorporating hepatic imaging, oxidative stress biomarkers, metabolomic analyses, and prespecified clinical endpoints will be important to clarify the biological significance of these observations and their potential relevance to metabolic disorders associated with T2DM [25].

### 4.5. Limitations

Several limitations of this study should be acknowledged. First, this was a post hoc analysis of a single-arm exploratory study with a relatively small sample size, thereby limiting statistical power and precluding definitive causal inference. In particular, the tendency for patients with higher baseline γ-GTP levels to exhibit greater change during treatments did not reach statistical significance and should therefore be interpreted as hypothesis-generating. Second, hepatic imaging, liver histology, and direct assessments of hepatic fat content or mitochondrial function were not performed. Consequently, the mechanistic basis linking γ-GTP, cholesterol metabolism, and mitochondrial regulation could not be directly evaluated. Third, serum γ-GTP may be influenced by alcohol consumption and other clinical factors [4]. Although additional analyses indicated that the reversible γ-GTP response was also observed in participants without habitual alcohol consumption, residual confounding cannot be completely excluded.

Despite these limitations, the study possesses several notable strengths. The prospective design with predefined treatment and withdrawal phases enabled detailed longitudinal evaluation of temporal changes at the individual patient level. Furthermore, the inclusion of a planned withdrawal period provided a unique opportunity to document the reversibility of γ-GTP changes, an aspect that has rarely been examined in previous studies of glucose-lowering agents. Although the observed association between γ-GTP and total cholesterol was derived from exploratory analyses, its consistency during both the treatment and withdrawal phases provides a basis for future hypothesis-driven investigations into the hepatometabolic effects of imeglimin.

## 5. Conclusions

In conclusion, γ-GTP levels showed a significant decrease during imeglimin treatment and increased during the post-treatment observation period in patients with T2DM, whereas AST and ALT exhibited only non-significant downward trends. Exploratory analyses identified total cholesterol as the only clinical parameter consistently associated with γ-GTP changes during both the treatment and post-treatment observation periods. Although these findings provide additional insight into the metabolic effects of imeglimin beyond glucose lowering, the exploratory analyses should be interpreted cautiously and require confirmation in larger prospective studies with prespecified endpoints and mechanistic assessments.

## Figures and Tables

**Table 1:** Clinical, metabolic, and hepatic parameters across the four study phases during and after imeglimin treatment.

Parameter	N	Baseline (−2–0 M)	Early TM (1–3 M)	Late TM (4–6 M)	Withdrawal (7–9 M)	*p* Value
FBG (mg/dL)	25	147.4 ± 22.7	133.4 ± 17.3 **	133.8 ± 22.5 *	154.3 ± 33.1 ^##^	<0.01
HbA1c (%)	25	7.4 ± 0.5	7.1 ± 0.5 **	6.9 ± 0.4 **	7.4 ± 0.7 ^##^	<0.01
GA (%)	25	18.5 ± 2.3	17.1 ± 2.3 **	16.7 ± 2.2 **	18.7 ± 3.0 ^##^	<0.01
BMI (kg/m^2^)	25	25.4 ± 3.1	25.3 ± 3.1	25.3 ± 3.2	25.3 ± 3.1	0.450
γ-GTP (U/L)	25	36.2 (27.1–48.4)	32.4 * (24.5–42.9)	31.1 * (23.5 –41.1)	35.6 ^#^ (26.4–48.5)	<0.01
AST (U/L)	25	21.8 (19.4–24.6)	21.7 (19.2–24.5)	21.9 (19.6–24.6)	23.1 (20.2–26.3)	0.249
ALT (U/L)	25	23.7 (19.0–29.6)	22.8 (18.2–28.6)	22.9 (18.4–28.5)	25.5 (19.6–33.1)	0.101
T-CHO (mg/dL)	25	188.9 ± 28.9	187.2 ± 28.7	185.2 ± 28.9	191.8 ± 32.8	0.240
HDL-C (mg/dL)	25	56.3 ± 12.1	56.2 ± 13.1	57.2 ± 12.9	57.4 ± 13.4	0.360
FTGs (mg/dL)	25	118.5 (99.6–140.9)	119.5 (101.9–140.2)	112.0 (94.4–132.9)	125.8 (105.0– 150.9)	0.074
TyG Index	25	9.06 ± 0.47	8.97 ± 0.43	8.90 ± 0.48	9.16 ± 0.47 ^##^	<0.01
FIB-4 Index	25	1.45 (1.15–1.83)	1.44 (1.15–1.82)	1.44 (1.15–1.82)	1.42 (1.13–1.78)	0.795

Data are presented as mean ± SD or geometric means with 95% CIs. * *p* < 0.05, ** *p* < 0.01 vs. Baseline Phase, ^#^ *p* < 0.05, ^##^ *p* < 0.01 vs. Late Treatment (TM) Phase. Statistical comparisons were performed using mixed-effects analysis followed by Tukey’s multiple comparisons test.

**Table 2:** Exploratory analyses of individual temporal response patterns for clinical and metabolic parameters.

Parameter	N	ΔTreatment vs. ΔWithdrawal	
		r	*p* Value
γ-GTP	25	−0.480	0.015
FBG	25	−0.027	0.899
HbA1c	25	0.183	0.381
GA	25	−0.144	0.493
BMI	25	−0.458	0.021
ASL	25	−0.090	0.668
ALT	25	0.002	0.993
T-CHO	25	−0.631	<0.001
HDL-C	25	−0.044	0.836
FTGs	25	−0.491	0.013
TyG Index	25	−0.448	0.025
FIB-4 Index	25	−0.391	0.053

Individual changes during treatment (ΔTreatment) were defined as the change from the Baseline Phase to the Late Treatment Phase, and changes after discontinuation (ΔWithdrawal) were defined as the change from the Late Treatment Phase to the Withdrawal Phase. Pearson’s correlation coefficients (r) and corresponding *p* values are shown. Variables with skewed distributions (γ-GTP, AST, ALT, fasting triglycerides, and the FIB-4 index) were analyzed after log10 transformation. These correlation analyses were performed as exploratory analyses to characterize individual response patterns and should be interpreted as hypothesis-generating rather than evidence of causal relationships.

**Table 3:** Associations between changes in γ-GTP and corresponding changes in clinical and metabolic parameters during the treatment and withdrawal phases.

Parameter	N	ΔTreatment (log γ-GTP)		ΔWithdrawal (log γ-GTP)	
		r	*p* Value	r	*p* Value
T-CHO	25	0.554	0.005	0.450	0.024
BMI	25	0. 449	0.024	0.355	0.081
FTGs	25	0.145	0.489	0.109	0.605
TyG Index	25	0.280	0.175	0.038	0.855

Change during treatment (ΔTreatment) was defined as the change from the Baseline Phase to the Late Treatment Phase, and changes after discontinuation (ΔWithdrawal) were defined as the change from the Late Treatment Phase to the Withdrawal Phase. Pearson’s correlation analyses were performed between ΔTreatment (log γ-GTP) or ΔWithdrawal (log γ-GTP) and the corresponding changes in clinical and metabolic parameters. γ-GTP and fasting triglycerides were analyzed after log10 transformation, whereas BMI, total cholesterol, and the TyG index were analyzed using their original values. Pearson’s correlation coefficients (r) and corresponding *p* values are presented.

## Data Availability

The data supporting the findings of this study are available from the corresponding author upon reasonable request. Due to ethical restrictions and participant confidentiality, access to the data may be limited and will be provided in accordance with institutional guidelines and applicable data-sharing policies.

## Supplementary Materials

The following supporting information can be downloaded at: https://www.mdpi.com/article/10.3390/jpm16080433/s1, Figure S1. Sensitivity analysis of longitudinal changes in serum liver enzymes, (a) γ-GTP, (b) AST, and (c) ALT, during and after imeglimin treatment using the full analysis set (FAS; n = 29). The green bar represents the 6-month imeglimin treatment period. The dashed horizontal line represents the baseline reference (0% change relative to Month 0). Values are presented as geometric means. Only the lower 95% confidence limits are shown for graphical clarity. * *p* < 0.05, ** *p* < 0.01 vs. Month 0, ^#^ *p* < 0.05 vs. Month 6. Statistical comparisons were performed using mixed-effects analysis followed by Tukey’s multiple comparisons test; Figure S2. Reversible changes in γ-GTP according to habitual alcohol consumption. Longitudinal changes in γ-GTP stratified by the presence or absence of habitual alcohol consumption (▲: Participants with habitual alcohol consumption, n = 16; ▼: Participants without habitual alcohol consumption, n = 9). The green bar represents the 6-month imeglimin treatment period. The dashed horizontal line represents the baseline reference (0% change relative to Month 0). Values are presented as geometric means. Only the lower 95% confidence limits are shown for graphical clarity.; Table S1: CONSORT 2010 Checklist for A Post Hoc Analysis of the INFINITY Study.

## Abbreviations

The following abbreviations are used in this manuscript:

ALT	Alanine aminotransferase
AST	Aspartate aminotransferase
BMI	Body mass index
CIs	Confidence intervals
FAS	Full analysis set
FBG	Fasting blood glucose
FTGs	Fasting triglycerides
FIB-4 Index	Fibrosis-4 Index
γ-GTP	γ-glutamyl transpeptidase
GLP-1RAs	Glucagon-like peptide-1 receptor agonists
GA	Glycoalbumin
GMR	Geometric mean ratio
HbA1c	Hemoglobin A1c
HDL-C	High-density lipoprotein cholesterol
MASH	Metabolic dysfunction-associated steatohepatitis
MASLD	Metabolic dysfunction-associated steatotic liver disease
PPS	Per-protocol set
SD	Standard deviation
T-CHO	Total cholesterol
TyG Index	Triglyceride–Glucose Index
T2DM	Type 2 diabetes mellitus
