# Supplementary material for: Imeglimin-Associated Temporal Changes in γ-Glutamyl Transferase and Total Cholesterol: A Post Hoc Exploratory Analysis of the INFINITY Study
Source: J Pers Med. 2026 Aug 17;16(8):433. doi: 10.3390/jpm16080433 (PMC13514984; doi:10.3390/jpm16080433)
Supplement: Supplementary file 1 [file jpm-16-00433-s001.zip › jpm-4437923-supplementary-Figures.pdf]

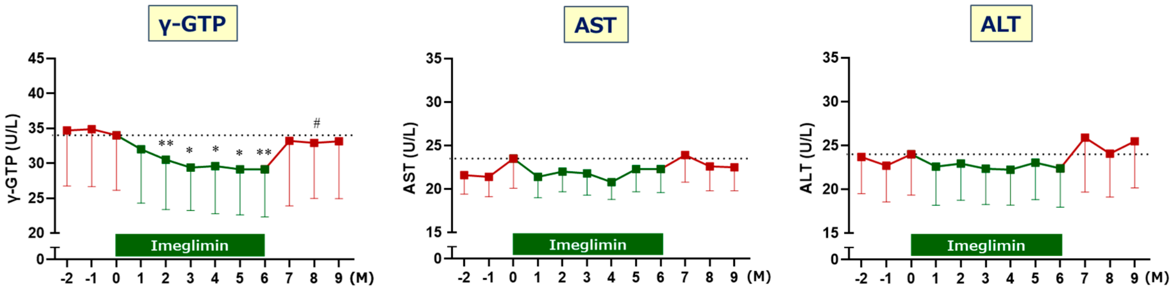

(**a**) (**b**) (**c**)

Supplemental Figure S1. Sensitivity analysis of longitudinal changes in serum liver enzymes, (a) γ-GTP, (b) AST, and (c) ALT, during and after imeglimin treatment using the full analysis set (FAS; n = 29). The green bar represents the 6-month imeglimin treatment period. The dashed horizontal line represents the baseline reference (0% change relative to Month 0). Values are presented as geometric means. Only the lower 95% confidence limits are shown for graphical clarity.

* P < 0.05, ** P < 0.01 vs. Month 0, # P < 0.05 vs. Month 6. Statistical comparisons were performed using mixed-effects analysis followed by Tukey’s multiple comparisons test.

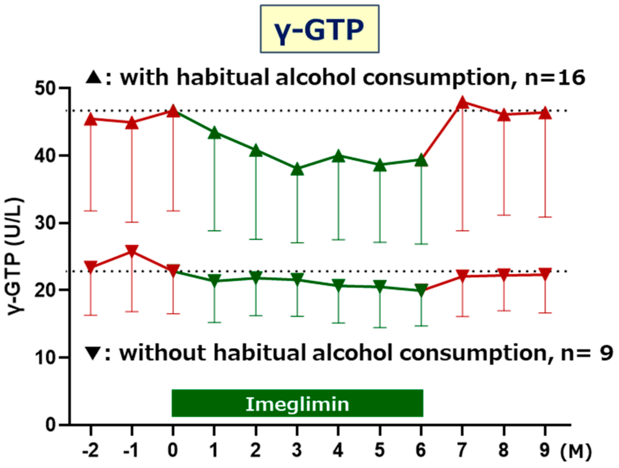

Supplemental Figure S2. Reversible changes in γ-GTP according to habitual alcohol consumption. Longitudinal changes in γ-GTP stratified by the presence or absence of habitual alcohol consumption (**▲:** Participants with habitual alcohol consumption, n=16; **▼**: Participants without habitual alcohol consumption, n=9). The green bar represents the 6-month imeglimin treatment period. The dashed horizontal line represents the baseline reference (0% change relative to Month 0). Values are presented as geometric means. Only the lower 95% confidence limits are shown for graphical clarity.
