# Supplementary material for: Imeglimin-Associated Temporal Changes in γ-Glutamyl Transferase and Total Cholesterol: A Post Hoc Exploratory Analysis of the INFINITY Study
Source: J Pers Med. 2026 Aug 17;16(8):433. doi: 10.3390/jpm16080433 (PMC13514984; doi:10.3390/jpm16080433)
Supplement: Supplementary file 1 [file jpm-16-00433-s001.zip › jpm-4437923-supplementary-Table S1.pdf]

CONSORT 2010 Checklist for A Post Hoc Analysis of the INFINITY Study

(Single-arm, open-label, prospective exploratory study; non-randomized)

Section/Topic	Item No	Checklist item	Reported on page/section
Title and abstract	1a	Identification as a randomized trial in the title	Not applicable (single-arm study)
	1b	Structured summary of trial design, methods, results, and conclusions (see CONSORT for abstracts)	Abstract
Introduction	2a	Scientific background and explanation of rationale	Introduction
	2b	Specific objectives or hypotheses	Introduction
Methods: Trial design	3a	Description of trial design (such as parallel, factorial) including allocation ratio	Methods: 2.1 Study Design
	3b	Important changes to methods after trial commencement, with reasons	N/A (Post hoc analysis)
Participants	4a	Eligibility criteria for participants	Methods: 2.1 Study Design, 2.2. Participants
	4b	Settings and locations where the data were collected	Methods: 2.1 Study Design
Interventions	5	The interventions for each group with sufficient details to allow replication	Methods: 2.1 Study Design
Outcomes	6a	Completely defined pre-specified primary and secondary outcome measures, including how and when they were assessed	N/A (Post hoc analysis)
	6b	Any changes to trial outcomes after the trial commenced, with reasons	Not applicable / none
Sample size	7a	How sample size was determined	N/A (Post hoc analysis)
	7b	When applicable, explanation of any interim analyses and stopping guidelines	N/A (Post hoc analysis)
Randomisatio n	8a	Method used to generate the random allocation sequence	N/A
	8b	Type of randomisation; details of any restriction (such as blocking and block size)	N/A
Allocation concealment mechanism	9	Mechanism used to implement the random allocation sequence	N/A
Implementati on	10	Who generated the allocation sequence, who enrolled participants, and who assigned participants to interventions	N/A
Blinding (masking)	11a	If done, who was blinded after assignment to interventions (e.g., participants, care providers, outcome assessors)	N/A

	11b	If relevant, description of the similarity of interventions	N/A
Statistical methods	12a	Statistical methods used to compare groups for primary and secondary outcomes	Methods – Statistical analysis
	12b	Methods for additional analyses, such as subgroup analyses and adjusted analyses	Methods – Statistical analysis
Results: Participant flow	13a	For each group, numbers of participants who were randomly assigned, received intended treatment, and were analysed	Methods: 2.2. Participants
	13b	For each group, losses and exclusions after randomisation, together with reasons	Methods: 2.2. Participants
Recruitment	14a	Dates defining the periods of recruitment and follow-up	N/A (Post hoc analysis)
	14b	Why the trial ended or was stopped	N/A (Post hoc analysis)
Baseline data	15	A table showing baseline demographic and clinical characteristics for each group	Results: 3.1. Baseline characteristics
Numbers analysed	16	For each group, number of participants included in each analysis and whether the analysis was by original assigned groups	Methods: 2.2. Participants
Outcomes and estimation	17a	For each primary and secondary outcome, results for each group, and the estimated effect size and its precision (e.g., 95% CI)	Results
	17b	For binary outcomes, presentation of both absolute and relative effect sizes	N/A
Ancillary analyses	18	Results of any other analyses performed, including subgroup analyses and adjusted analyses, distinguishing pre-specified from exploratory	Results
Harms	19	All important harms or unintended effects in each group	N/A (Post hoc analysis)
Discussion	20	Trial limitations, addressing sources of potential bias, imprecision, and, if relevant, multiplicity of analyses	Discussion – Limitations
	21	Generalisability (external validity, applicability) of the trial findings	Discussion
	22	Interpretation consistent with results, balancing benefits and harms, and considering other relevant evidence	Discussion
Other information	23	Registration number and name of trial registry	Institutional Review Board Statement, Title page
	24	Where the full trial protocol can be accessed, if available	Reference No. 15 to published protocol
	25	Sources of funding and other support; role of funders	Funding
